# Tumor Inflammatory Microenvironment of the Thyroid Cancer: Relationship between Regulatory T-Cell Imbalance, and p-NFΚB (p65) Expression—A Preliminary Study

**DOI:** 10.3390/jcm12216817

**Published:** 2023-10-28

**Authors:** Daniela Pasquali, Laura Giacomelli, Maria Carmela Pedicillo, Giovanni Conzo, Gabriella Gentile, Ilenia Sara De Stefano, Francesco Angelillis, Angela Santoro, Francesco Miele, Lucia Digitale Selvaggio, Rossella Melcarne, Giuseppe Pannone

**Affiliations:** 1 Department of Advanced Medical and Surgical Sciences, University of Campania “Luigi Vanvitelli”, 80100 Naples, Italy; lucia.digitaleselvaggio@unicampania.it; 2 Department of General and Specialist Surgery, Sapienza University of Rome, 00161 Rome, Italy; laura.giacomelli@uniroma1.it; 3 Department of Clinical and Experimental Medicine, University of Foggia, 71122 Foggia, Italy; mariacarmela.pedicillo@unifg.it (M.C.P.); ilenia_destefano.410008@unifg.it (I.S.D.S.); francesco_angelillis.506774@unifg.it (F.A.); giuseppe.pannone@unifg.it (G.P.); 4 Department of Experimental Medicine, University of Campania “Luigi Vanvitelli”, 80100 Naples, Italy; giovanni.conzo@unicampania.it; 5 Department of Radiology, Oncology and Pathology, Sapienza University of Rome, 00161 Rome, Italy; g.gentile@uniroma1.it; 6 General Pathology Unit, Department of Woman and Child’s Health and Public Health Sciences, Fondazione Policlinico Universitario Agostino Gemelli IRCCS, 00168 Rome, Italy; angela.santoro@policlinicogemelli.it; 7 General Surgery Unit, AOU Vanvitelli, 80100 Naples, Italy; francesco.miele@policliniconapoli.it; 8 Department of Translational and Precision Medicine, Sapienza University of Rome, 00161 Rome, Italy; rossella.melcarne@uniroma1.it

**Keywords:** thyroid cancer, Hashimoto’s thyroiditis, inflammatory microenvironment, CD25, NFkB

## Abstract

Background: Inflammatory microenvironment is an essential component of all tumors, including thyroid cancer. Autoimmune thyroid diseases are often associated with thyroid cancer. CD25, expressed in Treg cells and B cells, has been found to be associated with autoimmune thyroid diseases and the NFkB pathway is critical to tumor formation, regulating immune-related genes, and pro-inflammatory cytokine. Methods: Protein expression of CD25 and NFkB and its phosphorylated form was analyzed by immunohistochemistry in 80 patients with thyroid cancer (10 cases of cancers with Hashimoto’s thyroiditis and 70 cases without). Results: CD25 was mainly detected in the nucleus of the inflammatory cells such as in the thyrocytes and neoplastic cells. Protein staining was detected in the T-lymphocytes of the outermost zone of the lymphoid follicles. Moreover, in all cancer alterations, there were a higher level of p-NFkB than in the surrounding tissues. Again, p-NFkB staining was evident in neoplastic cells but not evident in inflammatory cells. Conclusions: Strong inflammatory infiltrate in the tumor microenvironment is correlated with an invasive phenotype. CD25 and p-NFkB levels were statistically significantly overexpressed in cancer cells.

## 1. Introduction

Differentiated thyroid cancer (DTC) claims thousands of lives worldwide each year and is the most common endocrinological malignancy. The Surveillance, Epidemiology, and End Results (SEER) database estimates that thyroid cancer will account for approximately 2.2% of all new cancer cases in the United States in 2023, with approximately 2120 deaths during the same period [1]. The highest increase in incidence is found in papillary thyroid cancer (PTC) [2]. The main risk factor predisposing to the onset of thyroid cancer is linked to exposure to ionizing radiation [3]. Other predisposing factors have been identified, such as a lack of iodine in the diet and heredity. The most frequent genetic alterations, identified by molecular biology studies during the past 20 years, are mainly those responsible for the activation of oncogenic genes such as BRAF, RET, NTRK1, SAR, and MET and the silencing of tumor suppressor genes such as p53, RASSF1A, PTEN, PPAR inhibitors of CDK [4]. The incidence of combined PTC, Hashimoto’s thyroiditis (HT), and Graves’ disease is increasing, but still undefined [5].

It has been estimated that up to 20% of all cancers develop from conditions of persistent inflammation such as autoimmune diseases or chronic infections [6]. PTC is often associated with organ-specific autoimmune diseases, such as HT, and RET/PTC, RAS, or BRAF activate a transcriptional program in inflammatory thyrocytes. Russell et al. have shown that the expression of RET/PTC3 in neoplastic cells induces an increase in the transcriptional activity of NFkB with a consequent increase in the secretion of proinflammatory cytokines CD25 [7]. CD25, the α chain of the receptor for interleukin 2 (IL-2Rα), is expressed in Treg cells and B cells [8]. The promoter of the CD25 gene contains more regulatory elements of DNA that bind to transcription factors key, NFkB and NF-AT, being involved in cancer progression [8]. However, the effect of HT on PTC prognosis and its possible mechanisms remains controversial [9]. IL-17A increases MHC class I expression and promotes T cell activation in papillary thyroid cancer patients with coexistent HT [9]. The primary purpose of this work is to study CD25 expression and the phosphorylated form of NFkB in thyroid cancer to evaluate a possible correlation with clinical and morphological parameters. We are also investigating whether differential expression of these markers in malignant epithelial tumors, benign epithelial tumors and HT may be useful as a prognostic factor in patients with thyroid cancer.

## 2. Materials and Methods

### 2.1. Patients and Methods

This study was conducted retrospectively on 80 cases of thyroid cancer (malignant epithelial neoplasms of the thyroid), 10 cases of HT, and 5 cases of adenoma, operated at the Department of General Surgery, University of Foggia, and diagnosed at the Section of Anatomic Pathology in the Department of Clinical and Experimental Medicine, University of Foggia, in the period between 2012 and 2019. All patients gave their written informed consent, and the study was approved by the Ethics Committee of the University of Campania “L. Vanvitelli” (AOU Università della Campania Vanvitelli–Prot. 0028416/i 23 November 2020). For each of the cases studied, biographical data, age, and sex were recorded. All cases were reviewed in blind by two expert pathologists (CP, AS), and for each tumor was defined the histological type in accordance with the criteria proposed by the TNM (AJCC-UICC 2018). Histological examination was carried out on samples taken during thyroid lobectomy with or without isthmectomy or total thyroidectomy. In all cases, the tumor was confined to the thyroid with or without lymph node metastases. For each patient, at least one of the samples taken during the sampling of surgical specimens included the tumor and the adjacent normal tissue. From the fragments fixed in 10% neutral buffered formalin and embedded in paraffin were obtained, for each case examined, at least 4 histological sections with a thickness of 3 microns. Some sections were collected on glass slides non-pre-treated with adhesive and then subjected to hematoxylin-eosin staining to confirm the histopathological diagnosis. Other sections were collected on polarized microscope slides for immunohistochemistry to evaluate the phenotypic expression of CD25 and p-NFkB. We used a mouse monoclonal anti-CD25 [Ventana CD25 (4C9)] and a polyclonal rabbit anti-p-NFkB [Santa Cruz Biotechnology, INC. Bergheimer, Heidelberg, Germany, p-NFkB p65 (Ser 536)]. The immunohistochemistry reaction was performed using the standard technique of immunoperoxidase on Benchmark XT, using diaminobenzidine (DAB) as chromogen and hematoxylin Gill’s type II as counterstain. To demonstrate the specificity of the antibodies used, negative control slides without primary antibodies were included for each color. The results of immunohistochemical staining were evaluated separately by two observers completely unaware of the histological typing and the follow-up of individual cases, in at least 10 fields at high magnification (HPF) using an optical microscope (Olympus BX41, to ×40, Olympus, Boston, MA, USA). The positive staining of the two antibodies has been assessed separately, in each case of cancer, tumor cells, thyrocytes, and peritumoral inflammatory cells. Immunohistochemical evaluation was performed also on 10 cases of HT, considering separately the thyrocytes by inflammatory cells, and on 5 cases of adenoma, considering separately adenoma cells from normal thyrocytes and by inflammatory cells. The staining pattern topography was also evaluated and recorded as cytoplasmic (C) and nuclear (N) and staining intensity was classified as weak (+, estimated to 40×), moderate (++, estimated to 20×), or strong (+++, valued at 10×). For each case, the cumulative percentage of positive cells among all sections was examined. The intra- and peri-tumor leukocyte infiltration was evaluated by assigning the following score: no inflammation 1: slight reaction and focal 2: moderate reaction 3: discrete reaction 4: reaction conspicuous and widespread. It was also specified the presence of intratumoral lymphocytes (TIL). Considering the immune cell population, for each case, two spots were chosen from representative areas of the lesion presenting important leucocyte infiltration, whereas two other spots were chosen from areas free of leucocytes. A morphological and immunohistochemical evaluation of CD25 in immune cells (TILs) has been performed for each tissue spot, estimating the number of positive cells per spot. In particular, the cases were grouped into categories for statistical analysis: negative (up to ten immune cells) and positive (more than 10 immune cells). In particular, the number of CD25+ infiltrating lymphocytes has been registered considering both intratumoral (intraepithelial lymphocytes infiltrating cell nests) and peritumoral areas (lymphocytes distributed in the infiltrate along the margin of the thyroid cancer). Patients were stratified into two groups by age ≤ 45 vs. >45 years in accordance with the criteria proposed by the TNM (AICC-UICC 2010). Inter-rater reliability between the two operating blinded and independent investigators who examined sections was assessed by immunohistochemical testing K of Cohen, obtaining values of K greater than 0.70 in almost all circumstances. 

### 2.2. Statistical Analysis

Clinical data were reviewed to record the sex, the age of patients and their clinicopathological parameters (TNM, goiter, HT, infiltration of the capsule and/or soft tissue, stage, growth mode, multifocal neoplasia, degree of inflammation and presence of TIL). The expression of CD25 and the p-NFkB were subjected to univariate statistical analysis to demonstrate possible associations with clinical-pathological data. To standardize our study population, we have removed some histological types present in the initial sample, because they represent numeric fractions too small to be studied by statistical analysis. Data were analyzed using Microsoft Excel 2007, version 12, and PRIMIT SOFA STATISTIC 2021, Version 1.5.4. Statistical evaluations were performed using the Student-Newman-Keuls test of linear correlation of Pearson, Spearman’s test, Kruskal-Wallis H test, and Mann-Whitney test.

## 3. Results

**Figure 1:**
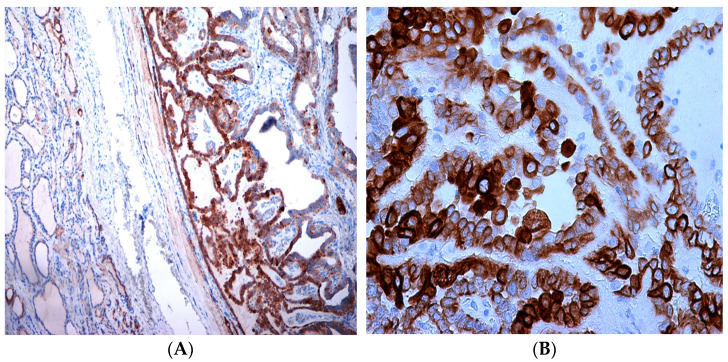
Immunohistochemical expression of p-NFkB in papillary thyroid cancer. (**A**) Note the intense cytoplasmic staining of neoplastic cells compared to cytoplasmic staining of peritumoral thyrocytes (4×). (**B**) Nuclear spot focal lengths (40×).

**Figure 2:**
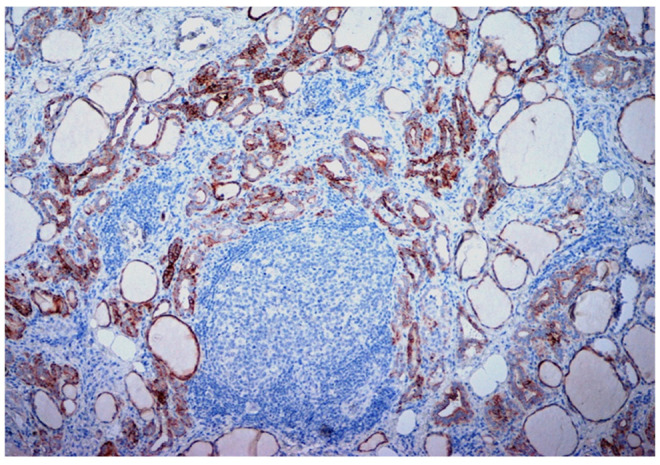
Immunohistochemical expression of p-NFkB in Hashimoto’s thyroiditis. Note the intense cytoplasmic staining of thyrocytes and absent expression for the same antibody in the germinal center (10×).

**Figure 3:**
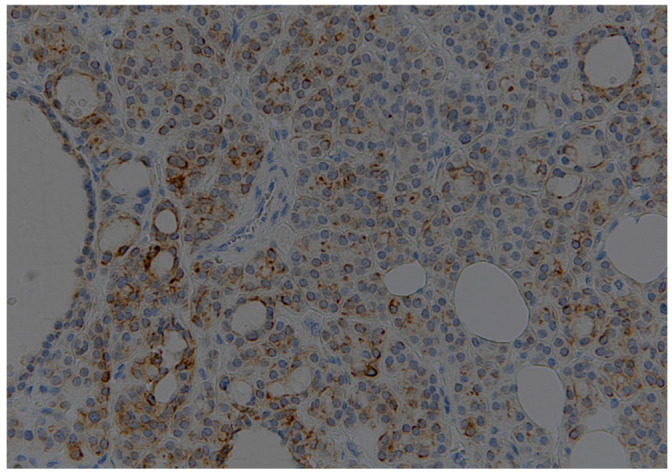
Thyroid adenoma; an immunohistochemical expression of p-NFkB (20×).

**Figure 4:**
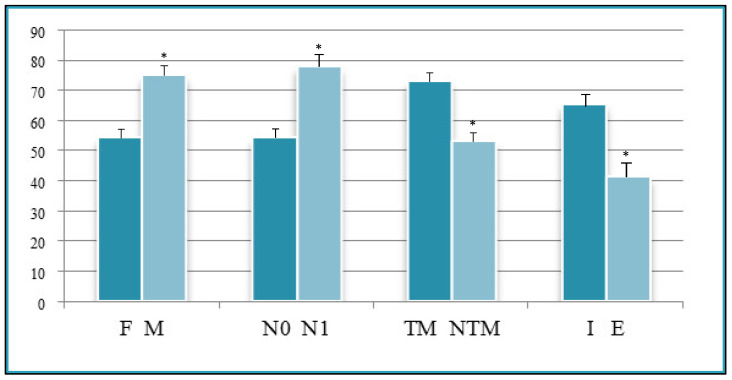
Univariate statistical analysis (ANOVA/*t*-test): cytoplasmic expression of p-NFkB and statistically significant parameters. F, females; M, males; N0, absence of metastases in regional lymph nodes; N1, metastases in regional lymph nodes; TM, soft tissue infiltration; NTM, absence of soft tissue infiltration; I, infiltrative phenotype; E, expansive phenotype. * *p* < 0.05.

**Figure 5:**
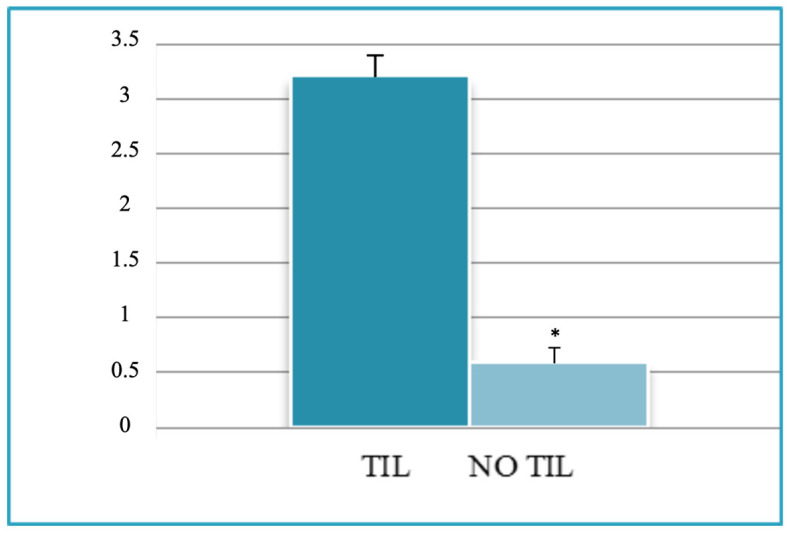
Univariate statistical analysis (ANOVA/*t*-test): nuclear expression of p-NFkB and presence of intratumoral lymphocytes. TIL, tumor-infiltrating lymphocytes. * *p* < 0.05.

**Figure 6:**
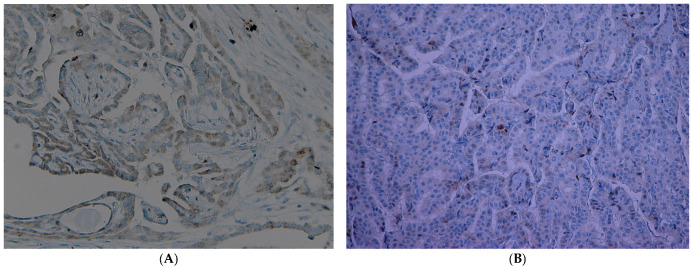
Immunohistochemical expression of CD25 in papillary thyroid cancer ((**A**) 20×, (**B**) 25×).

**Figure 7:**
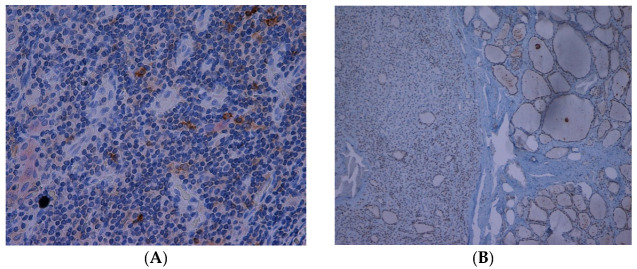
(**A**) Immunohistochemical expression of CD25 in Hashimoto’s thyroiditis (10×). (**B**) Immunohistochemical expression of CD25 in adenoma (10×).

**Figure 8:**
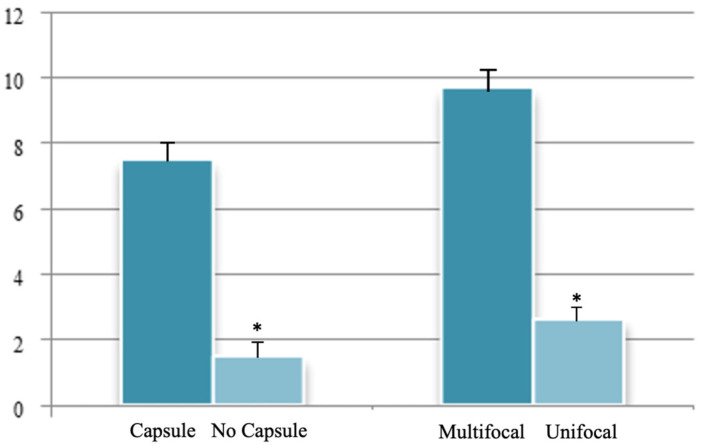
Univariate statistical analysis (ANOVA/*t*-test): nuclear expression of CD25 and statistically significant parameters. * *p* < 0.05.

**Figure 9:**
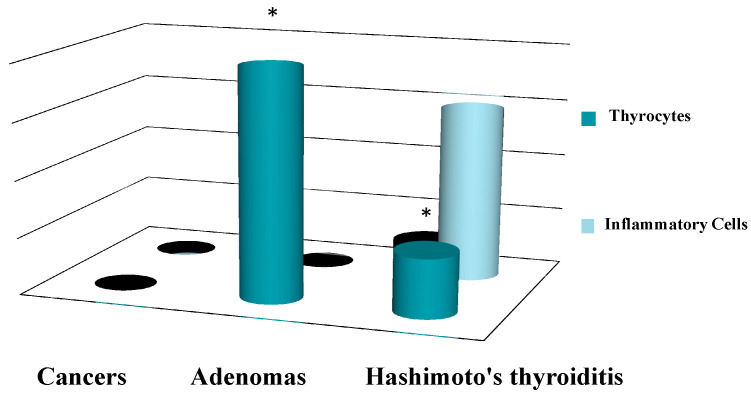
Kruskal-Wallis H-test: comparison of nuclear expression of CD25 in thyrocytes and inflammatory cells from the three patient groups. * *p* < 0.01.

A total of 95 patients were selected for the evaluation of CD25 and of p-NFkB, consisting of 75 PTC, 2 follicular carcinoma (variant Hürthle), 1 poorly differentiated carcinoma (insular variant), 2 anaplastic thyroid cancers (Table 1), 10 cases of HT, 4 follicular adenomas and 1 case of trabecular adenoma. The study, conducted on 80 thyroid cancer, included 19 (23.8%) male patients and 61 (76.2%) females (Table 1); 9 out of 10 patients with HT were female (90%) and 4 out of 5 patients with thyroid adenoma were male (80%). The age of cancer patients was between 24 and 95 years (mean 49.93 years ± SD 14.72) (Table 1); in patients with HT the age was between 29 and 59 years (mean 46.9 years ± SD 8.84); Finally, patients with thyroid adenoma were aged between 18 and 56 years (mean 40 years ± SD 18.83). No patient at the time of clinical diagnosis presented distant metastasis (Table 1). The histological review of 80 patients with thyroid cancer revealed that 12 (15%) had a concomitant HT; in 30 out of 80 patients (37.5%) was not observed inflammatory infiltrate, 20 out of 80 patients (25%) showed a slight and focal inflammation, 8 out of 80 patients (10%) a moderate inflammation, 11 out of 80 patients (13.75%) a discrete inflammation, and 11 out of 80 (13.75%) a substantial and widespread inflammation (Table 1). The immunohistochemistry results showed a substantial positive cytoplasmic p-NFkB staining (expressed as percentage counts: no positive cells/total cells) and only a focal nuclear expression in tumor cells (Table 2) (Figure 1). The peritumoral thyrocytes showed only cytoplasmic staining, while peri and intra-tumoral inflammatory cells were negative for the marker (Table 2). In the 10 samples of HT, we considered separately thyrocytes by inflammatory cells: the latter were negative to the marker while thyrocytes showed only a cytoplasmic positivity (Table 2) (Figure 2). In 5 adenomas, the p-NFkB was only focally positive in the cytoplasm and the nucleus of the adenoma cells, positive in the cytoplasm, but not in the nucleus of thyrocytes and absent in inflammatory cells (Table 2) (Figure 3). The cytoplasmic expression of p-NFkB correlates positively with the male sex, presence of lymph node metastases, infiltration of soft tissue, and infiltrative phenotype (*p* < 0.05) (Figure 4). The nuclear expression of p-NFkB correlates positively with the presence of intratumoral lymphocytes (TIL) (Figure 5) (*p* < 0.05). Immunohistochemistry showed also a positive immuno-cytoplasmic and nuclear CD25 antibody staining (measured as percentage counts: no positive cells/total cells) in tumor cells and a more conspicuous immunopositivity cytoplasmic than nuclear in peritumoral thyrocytes (Table 3) (Figure 6). Peri and intratumoral inflammatory elements were positive for this marker with higher positivity in the nuclei (Table 3). In the 10 samples of HT, we considered separate thyrocytes by inflammatory cells: both were focally positive in the nucleus and the cytoplasm (Table 3) (Figure 7A). In 5 adenomas, the CD25 showed positive staining in the cytoplasm and the nucleus of thyrocytes and focal staining in the nucleus of inflammatory cells (Table 3) (see Figure 7B). The nuclear expression of CD25 in tumor cells correlates with the neoplastic infiltration of the capsule and a multifocal growth mode (*p* < 0.05) (Figure 8). No correlation was observed between the cytoplasmic expression of the two markers in the cancer cells and the peritumoral tissue. Cytoplasmic p-NFkB was significantly higher in patients with thyroid cancer (*p* < 0.01). Nuclear CD25 expression in thyrocytes was significantly higher in patients with thyroid adenoma (*p* < 0.01), while in inflammatory cells was increased in patients with HT (*p* < 0.01), the latter result was confirmed at the Mann-Whitney test which showed an increase of this marker in HT compared to cancer (*p* < 0.01) (Figure 9). No significant statistical value has emerged for age, goiter, TNM stage, and histological concomitant of HT, in the group of patients with thyroid cancer.

By means of univariate statistical analysis, we have seen how the nuclear expression of CD25 in neoplastic cells correlates positively with the neoplastic infiltration of the capsule and a multifocal growth mode (*p* < 0.05).

## 4. Discussion

To the best of our knowledge, this is the first work in which combined expression of CD25 and p-NFkB has been described in a series of patients with thyroid carcinoma, and therefore no statistical comparison between the two antibodies is reported in the literature. The inflammation-cancer link can be seen as consisting of two pathways: an intrinsic pathway when it is driven by genetic alterations that cause both inflammation and neoplasia, and an extrinsic pathway, where inflammatory conditions promote cancer development. Key roles in inflammation-mediated tumor progression are played by transcription factors, cytokines, chemokines, and infiltrating leukocytes [10]. However, the role of these cells is complex; numerous studies have highlighted the pro-tumor activity of inflammation, while other evidence has shown that inflammation can support antitumor functions by promoting protection against cancer progression [11]. This paradox may be explained by the specific circuits expressed in the tumor microenvironment and the abundance and activated state of different cell types at the tumor site [12]. Macrophages and immature dendritic cells also accumulate in human thyroid tumors, both in the tumor stroma and at the advancing front, increased Tregs CD4+ CD25 infiltration in thyroid tissue has been correlated with an invasive phenotype [13]. The oncogenes activated in thyroid carcinomas, RET/PTC, RAS, and BRAF, by triggering the MAPK cascade, can induce a proinflammatory transcriptional program in thyrocytes, which mainly includes cytokines, chemokines, and their receptors [14]. In thyroid cancer, these molecules can act by both autocrine and paracrine mechanisms to support tumor cell proliferation, survival, and invasiveness by inducing remodeling of the tumor stroma through recruitment of inflammatory cells, immune cells, endothelial cells, and bone marrow-derived cells [13,15]. Based on these observations, we favor the notion that, at least in the overt stage, the growth and progression of thyroid cancer are positively influenced by two main components of inflammation, one dependent on the cells that are present in the tumor stroma, the other dependent on activation, in epithelial tumor cells, of specific oncoprotein-mediated signals. For these reasons, not only oncoproteins but “cancer-related inflammation” represents an important target for innovative diagnostic and therapeutic strategies in thyroid cancers. The inflammation-cancer relationship could be disrupted in several ways: (1) inhibition of signal transducers and transcription factors that mediate the survival and growth of malignant cells in response to inflammatory cytokines; (2) sequestration of chemokines and cytokines that recruit and sustain inflammatory cells in the tumor microenvironment; (3) depletion of immune and inflammatory cells that promote tumor development and progression, while sparing cell types and effector functions that support protective immune responses; (4) selective inhibition of tumor-promoting cytokines without effects on antitumorigenic cytokine expression. In support of this, a mast cell inhibitor, sodium cromoglycate (Cromolyn), strongly reduces the growth of thyroid cancer xenografts in immunodeficient mice [13]. Among the various pathways activated during inflammation, NFkB is a transcription factor that plays a primary role in both the regulation of immune response and inflammation, and in carcinogenesis, cell proliferation, and apoptosis. Because increased NFkB activity at least partially enhances the intrinsic radio- or chemo-resistance of thyroid cancer cells, several specific or nonspecific inhibitors of NFkB have been tested by in vitro and in vivo studies [16,17]. A super-suppressor mutant form of IkBα or a dominant-negative form of IKK can block the NFkB pathway and has led in experiments to a significant increase in the apoptotic response to ionizing radiation or drugs compared with control cell lines [18]. Among drugs known to be useful in cancer treatment, some may exert a nonspecific inhibitory effect on NFkB. Nonsteroidal anti-inflammatory drugs (NSAIDs) such as aspirin and sulindac sulfide have been shown to inhibit the initiation and/or progression of some cancers, including colorectal cancer [18]. At least to some extent, the antitumor effects of NSAIDs could be attributed to suppression of NFkB activity due to inhibition of IKK b-dependent phosphorylation of IkB [18]. Arsenic trioxide (ATO), which is clinically used to treat promyelocytic leukemia and multiple myeloma, proteasome inhibitors, flavonoids, cyclopentenone prostaglandins, and glucocorticoids also have inhibitory effects on NFkB [18]. Further studies, however, have found that the clinical use of NFkB inhibitors in cancer therapy has not been as effective as expected. For example, it has been shown that NFkB inhibition can delay cancer cell growth but does not necessarily lead to massive cell death in most solid tumors because of the coexistence of other, sometimes aberrant, anti-, or pro-apoptotic pathways independent of NFkB [19]. However, although an NFkB inhibitor per se may have moderate potential in inducing apoptosis, it could play an apoptosis-permissive role in cells treated with cytokines, chemotherapeutic drugs, or radiation, which otherwise induce NFkB activation by increasing resistance to the same treatments [18]. Consistent with what has been reported in the literature, the results of our study, despite the limited case series, confirm the presence of an inflammatory microenvironment in 62% of tumor cases and a higher prevalence of thyroid carcinoma and HT in the female sex (76.2% and 90%, respectively). Fifteen percent of patients with carcinoma had concomitant HT at the time of diagnosis, and consistent with reports in the literature, papillary thyroid carcinoma histotype histology was found in all these cases. The age of carcinoma onset consistent with national statistics is higher in adulthood (mean 49.9 ± 14.7 years). In our study, we evaluated the nuclear and cytoplasmic immunohistochemical expression of CD25 and the phosphorylated form of NFkB in thyroid carcinoma, thyroid adenoma, and HT. A correlation of both antibodies with an invasive phenotype in tumor cells and CD25 in peritumoral thyrocytes was shown. Moreover, p-NFkB does not appear to be expressed in inflammatory cells unlike CD25, which is expressed by such cells, and its immunopositivity correlates with a multifocal growth mode, in agreement with the role of inflammatory cells in tumor progression already found in the literature. Combined evaluation of both antibodies has not yet been described in the literature on case series of thyroid carcinoma, thyroid adenoma, and HT. In our study, we saw how the two markers have statistically significant expression in cancer cells but not in peritumoral thyrocytes and inflammatory cells, confirming the hypothesis that as NFkB expression increases, CD25 transcription increases but also that, conversely, the inflammatory microenvironment present in thyroid cancer supports NFkB-mediated carcinogenesis. By analyzing the three groups of patients, we also observed that p-NFkB is more highly expressed in patients with thyroid cancer than in patients with thyroid adenoma or HT. CD25, confirming its expression on the membrane of B-lymphocytes, T-lymphocytes, and macrophages, is more present in the group of patients with HT, a disease characterized, in fact, by a dense inflammatory infiltrate. In contrast to the findings of previous studies, in our analysis, the nuclear immunopositivity of CD25 in peritumoral thyrocytes shows statistically significant expression in the group of patients with thyroid adenoma [20]. Other authors have also described the nuclear localization of CD25 in thyroid cells, but the biological significance of this nuclear translocation remains to be deciphered [20]. Given the small size of our case series, we believe, therefore, that further studies on a larger case series of patients with thyroid adenoma could provide interesting results.

## 5. Conclusions

Based on a systematic review of the literature, the combined expression of CD25 and p-NFkB on a series of patients with thyroid cancer has not yet been described. Our results suggested the key role of CD25 and p-NFkB in inflammation-associated carcinogenesis. Increased expression of CD25 and aberrant activation of the NFkB in tumors can lead to increased cell proliferation, and expression of anti-apoptotic protein, promote invasiveness and metastasis, inhibit the immune response, and increase resistance to drugs. NFkB and CD25 may be biomarkers of great speculation and useful for prognostic and therapeutic treatment in patients with thyroid cancer. Therefore, we believe that the completion of the study in a large series could provide results of great speculative and practical interest both for prognostic and therapeutic goals.

## Figures and Tables

**Table 1:** Demographic and clinic-pathological characteristics of the study population with thyroid cancer.

Patients		80
**Age**	Mean ± Standard Deviation (range)	49.93 year ± DS 14.72 (24–95)
**Sex**	Female	61
	Male	19
**Histotype**		
	**Follicular carcinoma**	2
	Hürthle variant	2
	**Papillary carcinoma**	
	Classic type	46
	Follicular variant	27
	Oncocytic variant	1
	columnar cell variant	1
	**Poorly Differentiated Carcinoma**	
	Insular variant	1
	**Anaplastic Carcinoma**	2
	Total	80
**Unifocal/Multifocal**		
	Unifocal	64
	Multifocal	16
**p-TNM**		
	p-T1	53
	p-T2	2
	p-T3	22
	p-T4	3
	p-N0	63
	p-N1	17
	p-M0	80
	p-M1	0
**Struma**		
	Yes	37
	No	43
**Capsule infiltration**		
	Yes	34
	No	46
**Soft tissue infiltration**		
	Yes	25
	No	55
**Infiltrative/expansive phenotype**		
	Infiltrative	60
	Expansive	20
**Hashimoto’s thyroiditis**		
	Yes	12
	No	68
**Immune response to the tumor**		
Inflammatory infiltrate	0	30
	I (mild and focal length)	20
	II (moderate)	8
	III (discreet)	11
	IV (conspicuous and widespread)	11
Tumor-infiltrating lymphocytes (TIL)	TIL	28
	No TIL	52

**Table 2:** Immunohistochemical determination of p-NFkB in the three patient groups.

	Neoplastic Cells	Thyrocytes	Inflammatory Cells
Thyroid Cancer	+++c/focal n	+c/-n	-c/-n
HT	-	+c/-n	-c/-n
Adenomas	++c/focal n	+c/-n	-c/-n

**Table 3:** Immunohistochemical expression of CD25 in the three patient groups.

	Neoplastic Cells	Thyrocytes	Inflammatory Cells
Thyroid Cancer	+c/+n	+++c/+n	+c/+++n
HT	-	focal c/++n	focal c/+++n
Adenomas	++c/+n	+c/+n	-c/focal n

## Data Availability

The data presented in this study are available on request from the corresponding author. The data are not publicly available due to privacy or ethical restrictions.
